# Impact of Interleukin-6 Activation and Arthritis on Epidermal Growth Factor Receptor (EGFR) Activation in Sensory Neurons and the Spinal Cord

**DOI:** 10.3390/ijms25137168

**Published:** 2024-06-28

**Authors:** Anutosh Roy, Gisela Segond von Banchet, Fátima Gimeno-Ferrer, Christian König, Annett Eitner, Andrea Ebersberger, Matthias Ebbinghaus, Johannes Leuchtweis, Hans-Georg Schaible

**Affiliations:** 1 Institute of Physiology 1/Neurophysiology, Jena University Hospital, Friedrich-Schiller-University, 07743 Jena, Germany; anutosh.roy@med.uni-augsburg.de (A.R.); gisela.segond_von_banchet@med.uni-jena.de (G.S.v.B.); fatimagimeno7@gmail.com (F.G.-F.); christian.koenig@med.uni-jena.de (C.K.); andrea.ebersberger@med.uni-jena.de (A.E.); matthias.ebbinghaus@gmx.de (M.E.); johannes.leuchtweis@diakoneo.de (J.L.); 2 Department of Trauma, Hand and Reconstructive Surgery, Experimental Trauma Surgery, Jena University Hospital, Friedrich-Schiller-University, 07743 Jena, Germany; annett.eitner@med.uni-jena.de

**Keywords:** IL-6 signaling, soluble IL-6 receptor, EGF, EGFR, pain, spinal cord, DRG, arthritis pain, microglia, arthritis

## Abstract

In tumor cells, interleukin-6 (IL-6) signaling can lead to activation of the epidermal growth factor receptor (EGFR), which prolongs Stat3 activation. In the present experiments, we tested the hypothesis that IL-6 signaling activates EGFR signaling in peripheral and spinal nociception and examined whether EGFR localization and activation coincide with pain-related behaviors in arthritis. In vivo in anesthetized rats, spinal application of the EGFR receptor blocker gefitinib reduced the responses of spinal cord neurons to noxious joint stimulation, but only after spinal pretreatment with IL-6 and soluble IL-6 receptor. Using Western blots, we found that IL-6-induced Stat3 activation was reduced by gefitinib in microglial cells of the BV2 cell line, but not in cultured DRG neurons. Immunohistochemistry showed EGFR localization in most DRG neurons from normal rats, but significant downregulation in the acute and most painful arthritis phase. In the spinal cord of mice, EGFR was highly activated mainly in the chronic phase of inflammation, with localization in neurons. These data suggest that spinal IL-6 signaling may activate spinal EGFR signaling. Downregulation of EGFR in DRG neurons in acute arthritis may limit nociception, but pronounced delayed activation of EGFR in the spinal cord may be involved in chronic inflammatory pain.

## 1. Introduction

Interleukin-6 (IL-6) signaling contributes significantly to inflammation in musculoskeletal diseases [1,2,3,4,5,6] and musculoskeletal pain states [7,8,9]. In pain, IL-6 signaling is involved in both peripheral nociceptor [10,11,12] and spinal cord sensitization [13,14,15]. Sensitization describes the development of hyperexcitability of neurons. Sensitization of pain pathways enhances pain perception and is a hallmark of clinically relevant pain states. IL-6 contributes to both inflammatory and neuropathic pain [10,16,17,18,19]. Since neurons, like most cell types in the body, do not express the membrane-bound IL-6 receptor, the neuronal effect of IL-6 is mediated by IL-6-trans-signaling [14]. In this process, IL-6 forms complexes with the soluble IL-6 receptor (sIL-6R), that activate the ubiquitously present gp130 transduction unit in the cell membrane [20,21].

An important intracellular signaling pathway of IL-6 is the activation of Stat3. In tumor cells, IL-6-induced activation of Stat3 was increased [22] or maintained [23] by activation of the epidermal growth factor receptor (EGFR). This led to the concept that in tumor cells, IL-6 activates Stat3, which in turn leads to the release of IL-6. This, in a secondary process, leads to binding of EGFR to the IL-6 receptor and prolongation of Stat3 activity at high levels, mediated by EGFR stimulation [23]. Since there is evidence that EGFR has potential relevance in pain generation, nociceptive processes involving IL-6 signaling may also involve EGFR activation.

EGFR is a tyrosine kinase receptor that regulates the growth, survival, proliferation, and differentiation of fibroblasts and hepatocytes [24]. Aberrant EGFR signaling drives tumorigenesis and cancer progression [24,25]. Targeting EGFR reduces cancer progression and cancer pain, presumably by interfering with pain mechanisms [24]. To date, EGFR has primarily been implicated in pain regulation in models of neuropathy. In models of spinal nerve injuries (SNIs), chronic constriction injuries (CCIs), and spinal cord injuries, EGFR mRNA was upregulated in the dorsal root ganglia (DRGs). Systemic EGFR inhibitors reversed mechanical allodynia in SNIs and CCIs, and in complete Freund’s adjuvant-induced lesions [24].

EGFR is highly localized in normal DRGs [26,27,28]. EGFR localization in the spinal cord has been found to be very low, mainly present in astrocytes forming glia limitans [24,29], and in some studies, not present in spinal cord neurons [27,30]. However, our understanding of the role of EGFR in nociception is limited, and its role in arthritic pain has not been investigated.

The present experiments had two specific aims. The first was to test the hypothesis that the activation/sensitization of nociceptive cells by IL-6 signaling leads to EGFR activation. This was tested by recording from spinal cord neurons and by examining IL-6 and EGFR signaling in microglial cells and DRG neurons. The second goal was to study EGFR receptor localization in DRG neurons and in the spinal cord, both in healthy animals and in animals at different stages of arthritis. From the latter experiments, we expected to learn whether EGFR labeling and activation coincide with pain-related behaviors. In one set of experiments, we focused on EGFR labeling in the DRGs of rats in which we had recently studied pain in antigen-induced arthritis (AIA) [31]. Rodent AIA is a highly reproducible monoarticular inflammation with a rapidly developing, pronounced acute inflammatory phase lasting several days [31]. In the second set of experiments, we focused on spinal EGFR labeling using material from mice with glucose-6-phosphate isomerase (G6PI)-induced arthritis [32]. G6PI-induced arthritis is a symmetric polyarthritis with slow onset and a pronounced inflammatory phase of several weeks, for which we had recently studied pain-related behaviors over weeks [32].

## 2. Results

### 2.1. Effects of Gefitinib on Spinal Cord Neuron Responses to Knee Stimulation

**Figure 1:**
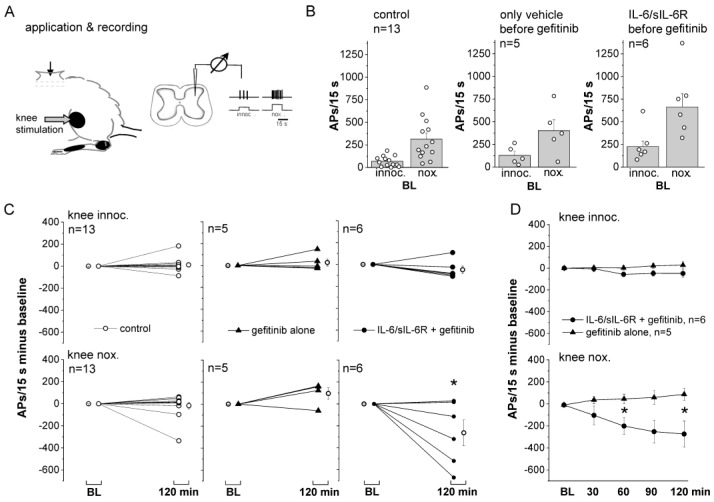
Effect of spinally applied gefitinib on the responses of spinal cord neurons to mechanical stimulation of the knee joint. (**A**) Experimental set-up. Spinal cord neurons with main input from the knee were recorded in the lumbar spinal cord. Stimulation was performed at innocuous and noxious intensities. (**B**) Average baseline (BL) responses of neurons of the control group (columns 1 and 2, no treatment), of neurons tested for the effect of gefitinib after the vehicle Tyrode (columns 3 and 4), and of neurons tested for the effect of gefitinib after pretreatment with IL-6/sIL-6R for 6 h (columns 5 and 6). (**C**) Left: no significant change from BL to innocuous and noxious stimulation in untreated neurons. Middle: no significant change from BL to innocuous and noxious stimulation by gefitinib alone. Right: no significant change from BL of responses to innocuous pressure and significant reduction from BL of responses to noxious pressure by gefitinib following pretreatment with IL-6/sIL-6R. * *p* < 0.05, one-sample *t*-test. (**D**) Comparison of changes of responses to innocuous (**top**) and noxious (**bottom**) pressure by gefitinib alone and gefitinib following spinal pre-administration of IL-6 and sIL-6R for 6 h. * *p* < 0.05, Mann–Whitney U-test. AP: action potential.

Gefitinib inhibits the EGFR family of tyrosine kinases by preventing the binding of ATP to the tyrosine domain of EGFR, thereby blocking EGFR activation [33]. We asked whether spinal application of gefitinib has an effect on the responses of spinal cord neurons with input from the knee joint to innocuous and noxious stimulation of the knee joint. Therefore, we performed in vivo extracellular recordings from spinal cord neurons in the lumbar spinal cord with input from the knee joint in anesthetized rats (Figure 1A, *n* = 24 rats, see Methods for selection of neurons). The neurons were located in the deep dorsal horn (953 ± 51 µm, mean ± SEM) and showed small responses to innocuous pressure and strong responses to noxious pressure applied to the knee (Figure 1B). Compounds were applied to a trough over the recording area. Control neurons (baseline responses: columns 1 and 2 in Figure 1B) were recorded for 2 h with the peripheral stimulation protocol but without spinal compound application. On average, they showed no significant change in response magnitude from the baseline response (Figure 1C, left). To test the effect of gefitinib, we first monitored the responses of neurons to knee stimulation with a vehicle (Tyrode) on the spinal cord (baseline: columns 3 and 4 in Figure 1B) and then applied gefitinib to the trough for 2 h. The responses to innocuous and noxious stimulation did not change significantly after switching from Tyrode to gefitinib (Figure 1C, middle). Thus, preventing EGFR activation did not affect the magnitude of spinal cord responses to stimulation in this time frame.

In the third group, we first applied IL-6 plus its soluble receptor (sIL-6R) to the spinal cord surface for 6 h prior to recording. Starting from the baseline (baseline: columns 5 and 6 in Figure 1B), we found that co-application of gefitinib to IL-6/sIL-6R complexes did not significantly alter the responses to innocuous stimulation, but significantly reduced the responses to noxious stimulation compared to the baseline (Figure 1C, right). Figure 1D shows the difference between gefitinib application after Tyrode only and after a preceding application of IL-6/sIL-6R. Gefitinib reduced the responses to noxious stimulation in the IL-6/sIL-6R-treated spinal cords but not in the Tyrode-treated spinal cords. These data suggest that the presence of IL-6 and sIL-6R and the induction of spinal cord hyperexcitability by IL-6-trans-signaling create a condition in which EGFR activation affects neuronal activity.

### 2.2. Effects of EGF, IL-6, and Gefitinib on BV2 Microglial Cell Activation

**Figure 2:**
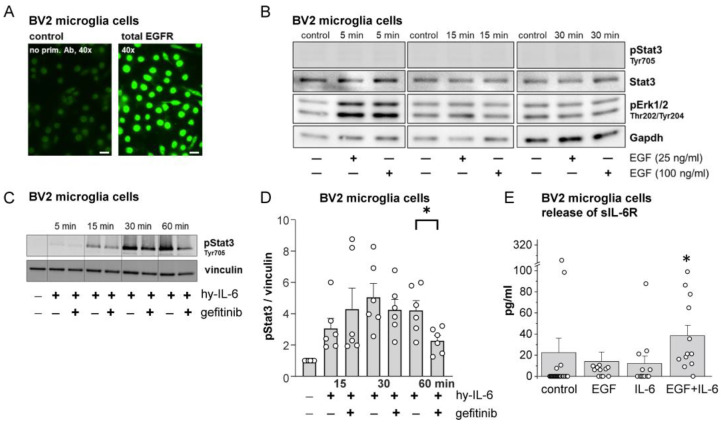
Effects of EGF, hyper-IL-6 (hy-IL-6, fusion of IL-6 and sIL-6R), and gefitinib on Stat3 and Erk1/2 signaling in BV2 microglial cells and release of sIL-6R. (**A**) Localization of EGFR in microglial cells. (**B**) Western blots showing transient Erk1/2 activation but no Stat3 activation by EGF at two concentrations. (**C**) Time-dependent Stat3 activation in microglial cells by hyper-IL-6 (50 ng/mL) and reduction of late Stat3 activation by coadministration of gefitinib (200 nM). (**D**) Densitometric analysis of experiments with the protocol shown in (**C**) *: *p* < 0.05, *n* = 6, Mann–Whitney U-test. (**E**) Release of sIL-6R from BV2 cells under control conditions, during EGF or IL-6 stimulation, or during co-stimulation with IL-6 (50 ng/mL) and EGF (50 ng/mL). *: *p* < 0.01, *n* = 12, post hoc Mann–Whitney U-test following Kruskal–Wallis test.

Microglial cells are critically involved in the generation of spinal hyperexcitability induced by IL-6/sIL-6R complexes. They have the membrane-bound IL-6 receptor [34], from which sIL-6R is cleaved and released, allowing IL-6-trans-signaling [14]. BV2 cells (a microglial cell line) express both membrane-bound IL-6R [35] and EGFR (Figure 2A), and release sIL-6R similarly to primary microglial cells [14]. Stimulation of BV2 cells with EGF immediately and transiently activated Erk1/2 but not Stat3 (Figure 2B). However, hyper-IL-6, a fusion molecule of IL-6 and sIL-6R [36], activated Stat3 (Figure 2C,D). We co-administered hyper-IL-6 and gefitinib for different time periods and monitored Stat3 activation. Figure 2C shows the progressive increase in pStat3 by hyper-IL-6 at 15 and 30 min with a stable high level of pStat3 after 30 min of hyper-IL-6 stimulation. The hyper-IL-6-induced pStat3 increase at 15 and 30 min was not significantly affected by coadministration of gefitinib, but the pStat3 activation at 60 min was significantly affected by coadministration of gefitinib. These results suggest that the initial pStat3 increase by hyper-IL-6 is not prevented by the EGFR blocker, but the late pStat3 increase has a component involving EGFR activation.

In further experiments, we measured the release of sIL-6R from microglial cells over a period of 1 h (Figure 2E). Only a few preparations showed sIL-6R release without stimulation. EGF or IL-6 slightly enhanced sIL-6R release, but co-application of EGF and IL-6 significantly increased sIL-6R release. Thus, co-activation of IL-6R and EGFR produces a greater effect than either IL-6R or EGFR activation alone.

### 2.3. Activation of EGFR in Cultured DRG Neurons by Hyper-IL-6 and EGF

**Figure 3:**
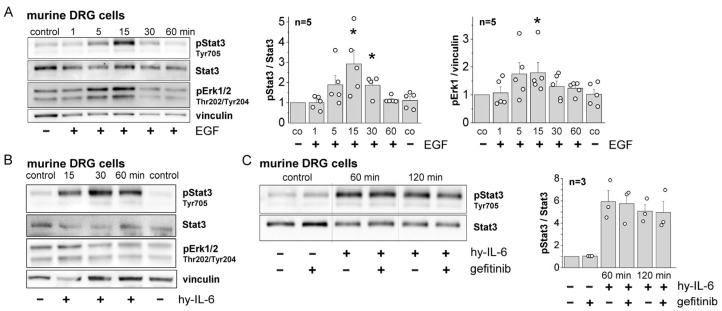
Effects of EGF, hyper-IL-6, and gefitinib on Stat3 and Erk1/2 signaling in cultured DRG neurons from mice. (**A**) Stat3 and Erk1/2 activation by EGF. *: *p* < 0.05, Mann–Whitney U-test with Bonferroni correction. (**B**) Stat3 and Erk1/2 activation by hyper-IL-6. (**C**) No reduction of hyper-IL-6-induced Stat3 activation when gefitinib was coapplied. Left: representative Western blot specimens, right: quantification of all data.

Since IL-6 sensitizes sensory neurons to mechanical stimulation (see Section 1) and EGFR is highly localized in normal DRGs, we also examined the effects of IL-6 and EGF on EGFR activation in DRG neurons. Western blots of cultured DRG neurons in Figure 3A show that stimulation of cultured DRG neurons with EGF caused phosphorylation of Stat3 (pStat3) and Erk1/2 (pErk1/2) within 5 min. Hyper-IL-6 caused strong Stat3 phosphorylation but no clear Erk1/2 phosphorylation (Figure 3B). Since both EGF and hyper-IL-6 caused Stat3 phosphorylation, we used this parameter to study IL-6/EGFR interaction. Co-application of gefitinib did not reduce hyper-IL-6-induced Stat3 activation in DRG neurons (Figure 3C). Thus, recruitment of EGFR signaling by preceding IL-6 signaling was observed in microglial cells and in recordings from spinal cord neurons, but not in DRG neurons. The latter were immediately activated by EGFR stimulation with the ligand EGF.

Taken together, these data support our hypothesis that IL-6 signaling can activate EGFR signaling, at least in the spinal cord. In DRG neurons, both IL-6 and EGF activate Stat3, but we found no evidence for an interaction between IL-6-signaling and EGFR activation.

In the second part of the project, we focused on the localization of EGFR in DRG neurons and in the spinal cord. We studied EGFR labeling and activation in normal animals and in two models of arthritis to gain insight into the potential dynamics of EGFR labeling and activation in a painful disease.

### 2.4. Labeling for EGFR and pEGFR in DRG Neurons from Normal Control Rats and Rats with AIA

We investigated the localization of EGFR and pEGFR in lumbar DRG neurons from normal rats and rats with AIA (*n* = 33 rats). AIA in rodents is a T-cell-mediated arthritis. In a first step, rats or mice are immunized against methylated bovine serum albumin (mBSA). Since this antigen binds to collagenous tissue in the joint and is retained at the site, injection of mBSA into a knee joint of immunized animals induces a highly reproducible monoarticular inflammation (similar pathology to acute human rheumatoid arthritis) with a uniform temporal course. It begins within 6 h after intra-articular antigen injection, reaches a stable peak on days 1–3, and then gradually resolves over the following weeks. We investigated whether the labeling for total EGFR and pEGFR in DRGs is altered in the course of AIA [31].

**Figure 4:**
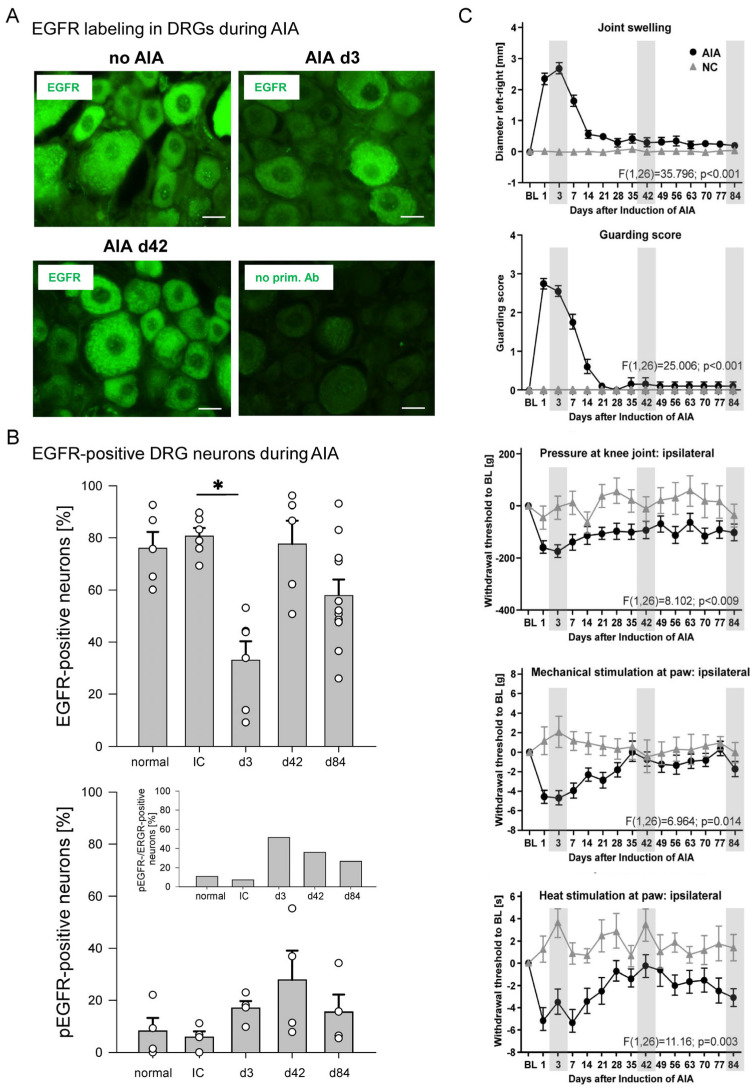
Localization of EGFR in DRG neurons of normal rats and of rats with AIA. (**A**) Representative specimens showing EGFR labeling in DRG sections of a healthy rat without AIA, of a rat at day 3 of AIA, of a rat at day 42 of AIA, and of a control section after omission of the antibody against EGFR. Scale bars 20 µm. (**B**) Proportions of DRG neurons labeled for EGFR (top) and activated EGFR (pEGFR, bottom) in normal rats, in immunized rats (IC), and rats at days 3, 42, and 84 of AIA. The inset compares the percentage of pEGFR-positive neurons with that of EGFR-positive neurons at these time points. *: *p* < 0.05, *t*-test (Bonferroni correction). (**C**) Pain-related behavior of rats in the course of AIA (modified from Leuchtweis et al., *Pain* **2020**, *161*, 1571–1583, from Figure 2 [31]). Each panel displays the pain-related behaviors of all rats in which AIA was induced (AIA, *n* = 20), compared with the pain-related behaviors of naive control rats (NC, *n* = 8). Note: EGFR labeling was only assessed in subsets of these rats; the particular animals were randomly selected. Gray boxes indicate the days for assessment of EGFR labeling. BL in (**C**), baseline value, normalized to zero. Values are mean ± SEM. Statistical analysis (AIA versus NC) in C: repeated-measures ANOVA, analysis of variance.

Figure 4A shows DRG sections from a normal rat (no AIA), a rat at day 3 of AIA, and a rat at day 42 of AIA, as well as a section processed without the primary antibody (no prim. Ab). We determined the proportion of neurons whose fluorescence intensity was greater than that of neurons not treated with the primary antibody. EGFR was localized in the vast majority of neurons from the normal rat, the immunized rat, and the rat at 42 days of AIA. However, EGFR labeling was much weaker in DRG neurons from the three-day AIA rats. Staining above background was found in 76.1 ± 6.2% (mean ± SEM) of neurons in sections from normal rats (normal, *n* = 5 rats), in 80.7 ± 8.1% of neurons from immunized rats (IC, *n* = 6), in 33.0 ± 7.2% of the neurons in sections from rats at day 3 of AIA (*n* = 6 rats), in 77.6 ± 9.0% of the neurons at day 42 of AIA (*n* = 5 rats), and in 57.9 ± 6.1% of the neurons at day 84 of AIA (*n* = 11 rats) (Figure 4B, top). Thus, labeling for total EGFR was observed in the vast majority of DRG neurons from normal and immunized rats, including neurons of all sizes, but EGFR intensity was significantly downregulated in DRG neurons three days after the onset of AIA (*p* = 0.016, *t*-test).

We also used an antibody against phosphorylated EGFR (pEGFR, see Supplementary Figure S1 and Section 2.5). In DRGs of normal rats and only immunized rats, less than 10% of DRG neurons were labeled for pEGFR. The proportion of pEGFR-positive neurons increased only slightly at 3 and 42 days of AIA (Figure 4B, bottom, *n* = 4). The inset to Figure 4B shows the proportion of pEGFR-positive neurons relative to the proportion of EGFR-positive neurons. While pEGFR labeling was found in less than 10% of EGFR-positive neurons in control and immunized rats, the proportion of pEGFR-positive neurons increased to approximately 50% of neurons with EGFR labeling on day 3 of AIA, and the proportion decreased during recovery from acute AIA. This pattern suggests that day 3 of AIA is characterized by EGFR downregulation, which may be associated with EGFR activation.

Figure 4C shows the pain-related behaviors in AIA [31] to which the EGFR labeling of the randomly selected animals must be related. EGFR localization was assessed on days 3, 42, and 84 of AIA (indicated by gray boxes). Compared to EGFR localization in naive control rats, EGFR localization was significantly reduced at day 3 (Figure 4B). At this time point, knee swelling was maximal, guarding score was at its peak, mechanical threshold decrease at the inflamed knee was maximal, mechanical and thermal thresholds at the ipsilateral paw were reduced (Figure 4C), and weight bearing was highly asymmetric [31]. Thus, on day 3 of AIA, rats exhibited the peak of inflammation and pain-related behaviors. By day 42, swelling had resolved, guarding score was similar to pre-inflammation, mechanical hyperalgesia at the knee was variable, mechanical threshold at the paw was similar to pre-inflammation, thermal threshold at the ipsilateral paw showed recovery (Figure 4C), and weight bearing was symmetrical (see [31]). At day 84, most parameters were similar to pre-inflammation, but some rats still showed a reduction in mechanical threshold at the knee (see [31]). Thus, the most striking finding was the downregulation of EGFR in DRGs during the acute, most severe phase of inflammation, when pain-related behaviors were maximal, and the recovery of EGFR labeling in DRG neurons as inflammation and pain subsided.

### 2.5. Labeling for EGFR and pEGFR in the Spinal Cord of Normal Mice and Mice with Chronic G6PI-Induced Arthritis

G6PI-induced arthritis is a symmetric polyarthritis with a slow onset and a duration of several weeks [32,37,38]. It is T- and B-cell-dependent and is induced by injection of purified G6PI in CFA into the tail of susceptible DGB/1 mice. Although G6PI is a ubiquitously expressed enzyme, immunization against G6PI causes a polyarthritis similar to human rheumatoid arthritis with activated immune cells and fibroblasts in the synovial membrane. The onset of manifest arthritis is 10 days after immunization, and the arthritis lasts for several weeks. Depletion of regulatory T cells prior to immunization exacerbates inflammation and bone destruction (more details below). Thus, in contrast to AIA, G6PI-induced arthritis has a pronounced chronic arthritic phase.

**Figure 5:**
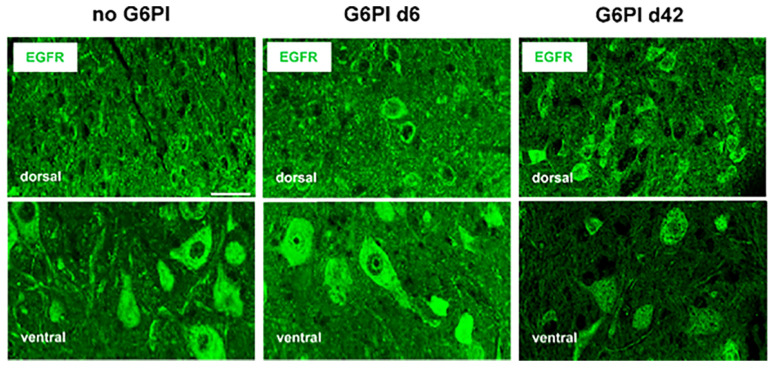
Labeling for EGFR in the spinal cord of normal mice and mice in the course of G6PI-induced arthritis. EGFR localization in the dorsal and ventral horn of mice without inflammation (no G6PI), of mice at day 6 of G6PI-induced arthritis (G6PI d6), and in the dorsal and ventral horn of mice at day 42 of G6PI-induced arthritis (G6PI d42). Mice were not depleted from Treg cells. Scale bar 10 µm.

Labeling for EGFR and pEGFR was performed in the spinal cord of 22 mice. Staining with an antibody against EGFR yielded the data shown in Figure 5 in the spinal cord. Similar positive labeling was observed in neuronal cell bodies in the spinal gray matter of the dorsal and ventral horn of normal mice (no G6PI) and in the spinal cords of mice on days 6 and 42 after induction of G6PI-induced arthritis. 

**Figure 6:**
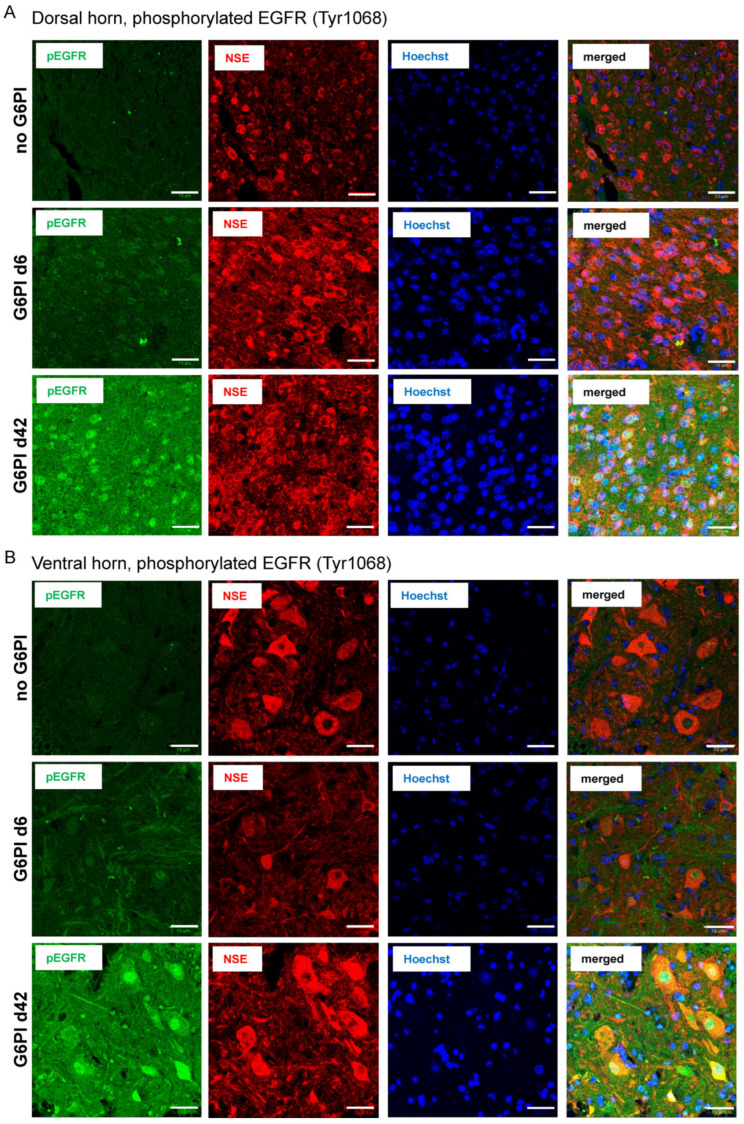
Localization of pEGFR and colocalization with neuronal markers in the spinal cord of mice in the course of G6PI-induced arthritis. (**A**) pEGFR (green), NSE (red), nuclear staining (Hoechst 34580, blue), and merged staining of pEGFR and NSE (yellow to orange) in the dorsal horn of mice without inflammation (no G6PI), in mice at day 6 of G6PI-induced arthritis (G6PI d6), and in mice at day 42 of G6PI-induced arthritis (G6PI d42). (**B**) pEGFR, NSE, Hoechst 34580, and merged staining in the ventral horn of mice without inflammation (no G6PI), in mice at day 6 of G6PI-induced arthritis (G6PI d6), and in mice at day 42 of G6PI-induced arthritis (G6PI d42). Mice were not depleted from Treg cells. Scale bar 10 µm.

**Figure 7:**
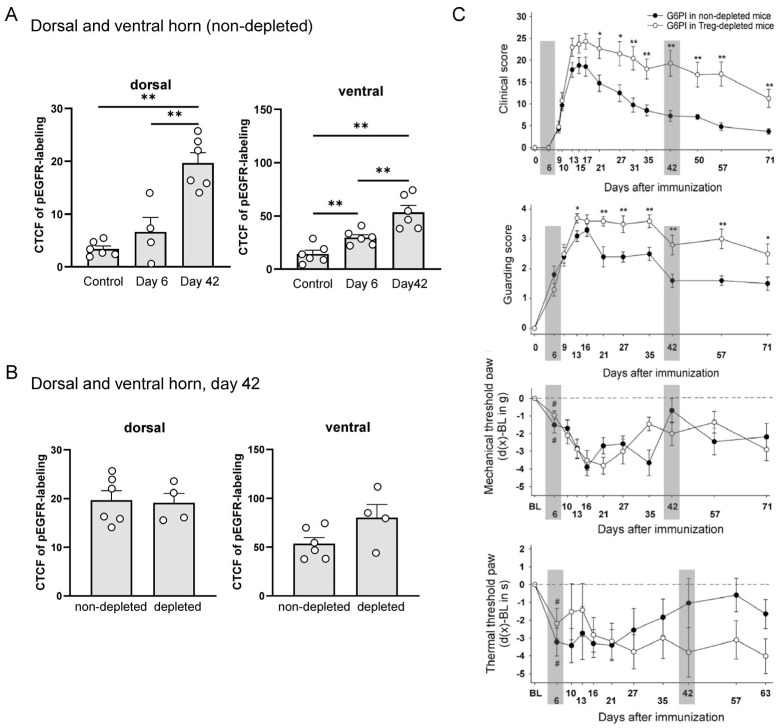
Labeling for pEGFR in the spinal cord of mice in the course of G6PI-induced arthritis and pain-related behaviors, in mice not depleted from Treg cells and in mice depleted from Treg cells. (**A**) Level of pEGFR labeling (CTCF: corrected total cell fluorescence) in the dorsal and ventral horn of non-depleted control mice, and of mice at day 6 and at day 42 of G6PI-induced arthritis. Each data point represents one animal. ** *p* < 0.01, Mann–Whitney U-test. (**B**) Expression level of pEGFR (CTCF) in the dorsal and ventral horn of Treg-depleted and non-depleted mice at day 42 of G6PI-induced arthritis. (**C**) Arthritis and pain-related behaviors in non-depleted and depleted mice in the course of G6PI-induced arthritis (data from Ebbinghaus et al., *Arthritis Rheumatol* **2019**, *71*, 2016–2026, from Figure 1 [32]). Clinical sore (swelling and erythema, *n* = 10 mice per group), guarding score (*n* = 10 mice per group), mechanical threshold at the paw (reduction of hind paw withdrawal threshold upon pressure, *n* = 18–26 mice per group), thermal threshold at the paw (reduction of hind paw withdrawal threshold upon heat application, *n* = 18–26 mice per group). Gray bars indicate the time points at which spinal cords were assessed for the pEGFR in randomly selected animals. Values are the mean ± SEM. * *p* < 0.05; ** *p* < 0.01 depleted versus non-depleted mice, by Student’s 2-tailed *t*-test; ^#^ *p* < 0.05 versus baseline, by Wilcoxon’s matched pairs signed rank test; first significant reduction of mechanical and thermal threshold at the inflamed hind paws compared to baseline (BL) values.

To test whether EGFR is activated during G6PI-induced arthritis, we then used an antibody against pEGFR (green) and performed colocalization studies with the neuronal marker Neuron-Specific Enolase (NSE, red) and cell nuclei (Hoechst 34580, blue), and merged for colocalization of pEGFR and NSE (last column) in the dorsal horn (Figure 6A) and ventral horn (Figure 6B). Only a few pEGFR spots were detected in sections from control animals (no G6PI). The pEGFR labeling increased slightly on day 6. However, strong labeling of pEGFR was found in neurons throughout the dorsal and ventral horn at day 42 (without primary antibodies, no labeling was observed; see Supplementary Figure 2), and the merged figures showed a strong overlap (yellow to orange) of pEGFR-positive (green) and NSE-positive cells (red). Figure 7A shows the quantification of pEGFR labeling of different animals (see specimens in Figure 6). Each data point in Figure 7 represents the average corrected total cell fluorescence (CTCF) of pEGFR labeling of one animal. These data suggest that EGFR was strongly activated in neuronal cells at day 42 of arthritis, but barely at an early time point (day 6).

Figure 7 relates pEGFR labeling to the expression of arthritis and pain-related behaviors, shown in Figure 7C (from Ebbinghaus et al. [32]). In Figure 7C, gray boxes indicate the time points at which pEGFR labeling data were analyzed. We randomly selected 4–6 animals per group. The inflammatory process and pain-related behaviors were examined in two groups of mice. “Non-depleted mice” were only immunized on day 0. In the second group, regulatory T (Treg) cells were depleted in vivo eleven and eight days before immunization on day 0. Depletion of Treg cells prior to immunization converts the self-limiting arthritis to a non-remitting arthritis with increased bone destruction [37,38]. Treg-depleted mice showed a higher clinical score (more swelling), a higher guarding score, and more persistent thermal hyperalgesia than non-depleted mice, but mechanical hyperalgesia in the paws was similar. Figure 7A shows that pEGFR was not significantly changed in the dorsal horn and only slightly but significantly increased in the ventral horn at day 6. At this time point, the clinical score was zero (no signs of inflammation), there was no bone destruction [32], but, most importantly, the guarding score was positive, and mechanical and thermal hyperalgesia were present. In addition, in the DRGs of mice at day 6 of arthritis, approximately 30% of the neurons showed activating transcription factor 3 (ATF3), a neuronal injury marker, whereas ATF3 was present in less than 3% of the DRG neurons of control mice [32]. Thus, the initial development of arthritic pain, which occurred before inflammation was manifest, was not associated with prominent spinal EGFR activation.

On day 42, mice showed marked inflammation, and Treg-depleted mice showed significant bone destruction. However, the guarding score and paw mechanical hyperalgesia were similar to day 6, and paw thermal hyperalgesia had already decreased. DRG neurons showed ATF3 similar to day 6 [32]. At day 42, EGFR was massively and significantly activated in the dorsal and ventral horn (Figure 7A). We also analyzed spinal EGFR activation in Treg-depleted mice at day 42 (Figure 7B) and found no difference between non-depleted and Treg-depleted mice. Thus, the more severe arthritis and bone destruction in Treg-depleted mice was not associated with greater spinal EGFR activation. 

**Figure 8:**
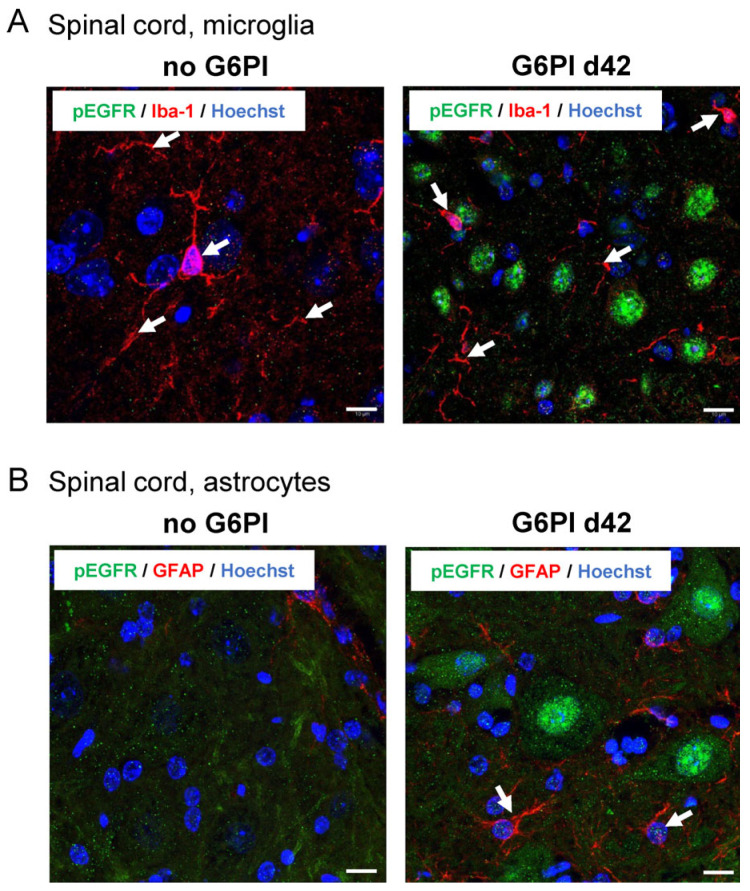
Representative images of pEGFR labeling in spinal cord microglia and astroglia (*n* = 3). (**A**) Spinal cord microglia (Iba-1, red) in the deep dorsal horn of control animals (no G6PI) and at day 42 of G6PI-induced arthritis (G6PI d42). At day 42, pEGFR (green) is not localized in microglia but in neurons. (**B**) Spinal astroglia in the ventral horn of control animals (no G6PI) and at day 42 of G6PI-induced arthritis (G6PI d42). No GFAP-positive activated astrocytes (red) and no pEGFR (green) in control animals (no GPI), and no pEGFR in GFAP-positive astrocytes at day 42 of G6PI-induced arthritis (G6PI d42). Arrows: examples of positively labeled cells, Scale bar 10 µm.

We also examined the localization of activated EGFR in microglial cells by co-labeling with Iba-1 and in astroglia by co-staining with GFAP. In the absence of inflammation (no G6PI), Iba-1-positive microglial cells appeared in red, while no pEGFR staining (green) was observed (Figure 8A, left). At day 42, Iba-1-positive branches were observed, but staining for pEGFR (green) did not overlap with Iba-1-positive structures (Figure 8A, right). In control mice, no GFAP (red) was detected because the GFAP antibody labels only activated astroglia in mice [39], and no pEGFR staining was observed (Figure 8B, left). At day 42, GFAP was clearly visible, but staining for pEGFR did not overlap with GFAP staining (Figure 8B, right). These data showed that pEGFR was not present in microglial and astroglial cells, but rather in neurons. 

## 3. Discussion

The present study tested the hypothesis that IL-6-induced signaling may involve activation of EGFR in the spinal cord and DRG neurons. Application of the EGFR activation blocker gefitinib to the spinal cord reduced neuronal responses to mechanical joint stimulation after application of IL-6 and sIL-6R for 6 h. In microglial cells, Stat3 activation by hyper-IL-6 was attenuated in a slow time course by gefitinib. The study showed high labeling for EGFR in DRG neurons. However, the labeling was significantly reduced in the acute phase of antigen-induced arthritis, which is characterized by significant mechanical and thermal hyperalgesia. This reduction was observed to be reversed in the phase of residual inflammation and normalization of pain-related behaviors. In a study of G6PI-induced arthritis in mice, spinal EGFR was observed to be barely activated during the initial pain phase of arthritis, but significantly activated during the chronic phase of arthritis. At this stage, pEGFR was identified in dorsal and ventral horn neurons, but not in microglia or astroglia. These data demonstrate that IL-6 signaling can activate EGFR signaling in the spinal cord and provide initial insights into the temporal dynamics of localization and activation of EGFR in models of arthritis.

We previously found that during the development of acute joint inflammation, IL-6 is released in the spinal cord and that spinal IL-6 signaling contributes to the generation of inflammation-induced spinal hyperexcitability because sgp130, which prevents IL-6-trans-signaling, attenuates the development of spinal hyperexcitability [15]. Later, we found that spinal IL-6 does not induce spinal hyperexcitability when microglial activation is blocked by minocycline [14]. Microglial cells provide sIL-6R, which forms complexes with IL-6 to allow IL-6-trans-signaling and activation of cells that do not have the membrane-bound IL-6 receptor, such as neurons. Thus, microglial cells are critically involved in the induction of spinal IL-6 effects. Since spinal IL-6/sIL-6R complexes are activated downstream of TNF [14] and IL-1β [40], IL-6-trans-signaling plays a key role in the generation of spinal hyperexcitability by cytokines, and microglial cells are essential in this process. Since IL-6 and EGF co-application evoked more sIL-6R release than either IL-6 or EGF alone, EGFR may be co-involved in microglial activation.

Since gefitinib did not reduce spinal responses to noxious stimulation in the naive spinal cord, EGFR does not appear to be involved in the basic responses of spinal neurons to noxious stimulation. The reduction of noxious stimulation responses by gefitinib after spinal pretreatment with IL-6/sIL-6R complexes suggests that IL-6-trans-signaling induces EGFR activation. This is supported by the study of microglial cells. Gefitinib reduced the effect of hyper-IL-6 on Stat3 activation at 60 min, but not at 15 and 30 min, suggesting that the late phase of IL-6-induced signaling involves EGFR activation which stabilizes Stat3 activation. This is reminiscent of the findings of Wang et al. [23], who found that the second wave of pStat3 increase (about 4 h after IL-6) is produced by EGFR activation. They proposed that EGFR associates with gp130, which is initially activated by IL-6. High IL-6 production and stimulation of gp130 then promotes EGFR recruitment. Erlotinib blocks EGFR phosphorylation and the second wave of pStat3 increase, which can no longer be prevented by SOCS3 activation, which blocks JAK1 and JAK2 [23]. Functionally, we found that activation of both IL-6R and EGFR by specific ligands (IL-6 and EGF) increased the release of sIL-6R from microglial cells more than either IL-6 or EGF alone. These data suggest that coactivation of both receptors enhances the release of sIL-6R from microglial cells.

In DRG neurons, both hyper-IL-6 and EGF stimulation activated Stat3 and Erk1/2, but we were unable to show that IL-6-induced Stat3 activation was affected by blocking EGFR activation. Thus, DRG neurons that do not have the membrane-bound IL-6 receptor may not exhibit this amplification mechanism, and there may be no interaction of IL-6 signaling and EGFR activation.

The difference between IL-6 and EGFR interaction in DRG neurons and in microglial cells prompted us to investigate the localization and activation of EGFR in both DRG neurons and in the spinal cord. We tested the hypothesis that EGFR is activated during arthritis, which is characterized by persistent mechanical hyperalgesia. We analyzed the localization and activation of EGFR in the DRGs and spinal cord in arthritis models in which we had previously extensively characterized pain behavior. This completely unbiased approach should provide further evidence for the involvement of EGFR activation in arthritis pain.

Consistent with previous studies in human, rat, and mouse DRGs [26,27,41,42], DRG neurons from normal control rats showed high levels of EGFR localization. A small proportion of DRG neurons showed pEGFR, suggesting that EGFR is rather inactive under normal conditions. However, labeling of EGFR with an antibody that recognizes total (activated and inactivated) EGFR, showed significant downregulation of EGFR during the phase of highest acute inflammation and strongest pain-related behaviors, and a recovery during the phase of decreasing inflammation and pain. Thus, maintenance of severe acute arthritis pain was not critically dependent on sustained high levels of EGFR in DRG neurons. Immunization, which does not induce pain-related behaviors in rat AIA [31], did not affect EGFR labeling. In general, EGFR is internalized and recycled to the plasma membrane at low ligand concentrations but is not recycled and degraded by ubiquitination at high ligand concentrations [28,43]. Signaling continues until the receptors are either recycled back to the cell surface or engulfed in proteolytic lysosomes [28]. The fact that a higher proportion of the remaining EGFR was activated on day 3 of AIA suggests that EGFR activation may have led to EGFR degradation. EGFR may have been activated by specific ligands, such as epiregulin (EREG) [44]. Furthermore, EGFR may even be activated by G protein-coupled receptor (GPCR) stimulation, where EGFR transactivation is mediated by the tyrosine kinases Src and Pyk, downstream of GPCR activation [45]. Because we were unable to show that IL-6-induced Stat3 activation in DRG neurons was affected by the EGFR blockade, IL-6 may not play a role in EGFR activation in DRG neurons, although IL-6 is important for the development of swelling and mechanical hyperalgesia in AIA [46].

Because EGFR stimulation induces pronociceptive effects in DRG neurons, such as potentiation of TRPV1-dependent calcium currents [27], EGFR downregulation in DRG neurons may limit nociception. Verma et al. [44] reported that EREG, a ligand of EGFR in humans, may protect against sensitization to acute pain. Such an effect may result from EGFR downregulation after stimulation. However, the peripheral and spinal nociceptive effects of EGFR may be opposite.

Although spinal EGFR activation can occur within several hours (see above) the activation pattern of EGFR in G6PI-induced arthritis suggests that EGFR activation may be particularly important in chronic arthritis. Although the severity of pain-related behaviors was comparable on day 6 and day 42 after immunization, EGFR activation was weak on day 6 and very pronounced on day 42, which is characterized by prolonged inflammation and persistent pain. However, the higher clinical score, more severe bone resorption, and more severe thermal hyperalgesia in Treg-depleted mice at day 42 were not reflected in further EGFR activation.

Notably, at day 42 of arthritis, highly activated EGFR was observed in neurons and not in microglial and astroglial cells. Thus, chronic arthritis may upregulate neuronal pEGFR in particular. EGFR plays an important role during brain development and is then downregulated [28,47,48]. In the adult CNS, astrocytes barely show EGFR 2-3 months after birth and remain as quiescent astrocytes, and neurons may show basal EGFR levels [25]. EGFR, especially pEGFR, is upregulated after neuronal injury and in pathological conditions such as Parkinson’s, Huntington’s and Alzheimer disease, and amyotrophic lateral sclerosis (ALS) [25,28]. In these conditions, EGFR may have neuroprotective effects, but strong EGFR activation in astroglial cells inhibits regeneration after injury, such as spinal cord injury [30,49,50]. Whether EGFR inhibitors or, rather, EGF restore function is controversially discussed [28,51,52].

The high pEGFR localization in neurons but not in astrocytes and microglia at day 42 of arthritis does not suggest an EGFR activation pattern typical of injury and neurodegeneration. EGFR may be involved in persistent neuronal hyperexcitability. In cultured hippocampal EGFR-positive neurons, EGF enhanced NMDA-mediated increases in intracellular Ca^2+^ concentration [53] and increased long-term potentiation (LTP) in rat hippocampal slices and in the hippocampus in vivo [54,55]. NMDA receptors are involved in acute and chronic arthritis-induced spinal hyperexcitability [56]. Furthermore, since spinal prostaglandins are involved in inflammatory pain, EGFR may be involved in neuronal cyclooxygenase-2 (COX-2) upregulation in the spinal cord [56]. Similar to other tyrosine kinase receptors, EGFR can be translocated to the nucleus through a nuclear import system associated with the nuclear pore complex, and act as a transcription factor [57,58]. In cancer cells, this can activate the inducible nitric oxide synthase (iNOS), and COX-2 genes at the transcriptional level [59,60]. Taken together, such effects may support the development of chronic pain as suggested by Verma et al. [44]. Whether neuroprotective mechanisms are activated by EGFR stimulation in chronically challenged spinal cord neurons remains to be investigated. The substantial EGFR activation in both dorsal and ventral horn neurons is suggestive of a more general response of the spinal cord during chronic arthritis.

In conclusion, the present data show several salient findings. First, in the spinal cord, IL-6 signaling can activate EGFR signaling. Using recordings from spinal cord neurons, we were able to show that spinal EGF signaling comes into play when the spinal cord is stimulated by IL-6-trans-signaling, a mechanism involved in the generation of spinal hyperexcitability, e.g., during the development of arthritis. Microglial cells have been identified as a site of IL-6/EGF interaction. They play a key role in the generation of spinal hyperexcitability by releasing sIL-6R, which is required for the process of IL-6-trans-signaling. Here we found that the combined application of both IL-6 and EGF stimulated the release of sIL-6R from microglial cells more than either IL-6 or EGF alone. Since the induction of spinal hyperexcitability is an important mechanism of central pain amplification, spinal EGFR activation adds to the mechanisms of induction of central sensitization [56]. An IL-6/EGF interaction could not be demonstrated for DRG neurons.

Second, we obtained detailed insights into the dynamics of EGFR localization and activation in DRG neurons and in the spinal cord during the course of arthritis. DRG neurons in normal rats showed a high level of EGFR localization. However, during the acute and most painful phase of arthritis, EGFR was downregulated in DRG neurons, suggesting that EGFR activation in peripheral sensory neurons is not a critical neuronal mechanism for the maintenance of severe pain. EGFR labeling recovered as inflammation and pain subsided. In the spinal cord of mice, little neuronal EGFR activation was observed in the early painful stages of arthritis, but strong EGFR activation was found in the chronic painful stage of arthritis. Since pEGFR labeling in chronic arthritis was found in both dorsal and ventral horn neurons, widespread neuronal pEGFR activation in the spinal cord was identified as a signature of chronic pain. This pEGFR activation may strongly support spinal mechanisms of persistent arthritic pain, as EGFR activation was found to be associated with several neuronal mechanisms of hyperexcitability. The present data invite investigation of whether inhibition of spinal EGFR activation may be a target for the treatment of chronic pain states in arthritis, which are sometimes difficult to treat with non-steroidal analgesics and cytokine neutralization.

## 4. Materials and Methods

### 4.1. Animal Experiments

All animal experiments were approved by the Thuringian State Office for Food Safety and Consumer Protection, Consumer Health Protection Division (Thüringer Landesamt für Verbraucherschutz, Reg. Nr. 02-071/16) and were performed in strict accordance with the European Community guidelines for the care and use of laboratory animals. Data collection, analysis, and presentation were in accordance with the ARRIVE guidelines. All injections for immunization, and induction of arthritis were performed under brief anesthesia with 2.5% (rats) or 5% (mice) isoflurane (LKT Laboratories, Saint Paul, Minnesota, USA). Isoflurane was administered through a face mask.

### 4.2. Recordings from Spinal Cord Neurons In Vivo

Male rats were deeply anesthetized with sodium thiopental (100–120 mg/kg i.p., Rotexmedica, Trittau, Germany). The trachea was cannulated, and a gentle stream of oxygen was blown towards the cannula to assist respiration. Catheters were inserted into the carotid artery to measure mean arterial blood pressure (usually approximately 100 ± 20 mm Hg) and into the jugular vein. The catheters were filled with Tyrode’s solution (140 mM NaCl, 2.7 mM KCl, 2.4 mM CaCl_2_, 2.3 mM MgCl_2_, 12 mM NaHCO_3_, 0.24 mM NaH_2_PO_4_, pH 7.4) and heparin (12.5 I. U. for the arterial catheter and 1 I. U. for the catheter in the vein, Heparin-Natrium-25000, Ratiopharm, Ulm, Germany). Body temperature was maintained at 37 °C with a feedback thermostat. If necessary, additional injections of sodium thiopental (12 mg/kg i.p.) were used to maintain a deep level of anesthesia characterized by stable blood pressure during noxious stimulation and absence of corneal reflexes.

The animal was fixed in a frame, and the lumbar segments L1-L4 were exposed by laminectomy. The dura mater was opened. The surgical area was stabilized with 3% agar in Tyrode’s solution, leaving a tightly sealed trough (3 × 5 mm) on the surface of the spinal cord, which was immediately filled with approximately 50 µL Tyrode’s solution to avoid tissue desiccation. For application of gefitinib, IL-6, and sIL-6R to the spinal cord, the Tyrode’s solution was removed from the trough and replaced with the compounds to be used for testing.

Extracellular recordings from single neurons with input from the knee joint were made with glass-insulated carbon filaments. For data collection, neurons were selected that responded to pressure applied to the ipsilateral left knee joint but did not respond to stroking and squeezing of the overlying skin. Such neurons are typically found in the deep dorsal horn. The total receptive field in the leg was determined. Action potentials from one neuron were continuously monitored on a digital oscilloscope and stored for final off-line spike analysis using Dapsys 8.42 software [61] or Spike/Spidi software [62].

To test neuronal responses, mechanical stimuli were applied to the mediolateral axis of the knee with a manometer (Correx, Koeniz, Switzerland) at innocuous (1.9 N/40 mm^2^) and noxious (7.8 or 5.9 N/40 mm^2^) intensities. Subsequently, the ankle and paw were stimulated with defined clamps (innocuous: 1.1 N/20 mm^2^, noxious: 5.8 N/20 mm^2^). Each stimulus lasted for 15 s, followed by 15 s of no stimulation. The whole stimulation cycle was repeated every 5 min.

Once a suitable neuron was identified, the specific recording protocol was initiated. In one group of rats (control neurons) we just repeated the stimulation procedure for two hours. In another group of rats, we measured baseline responses to innocuous and noxious stimulation for 30 min, then we replaced the vehicle Tyrode with gefitinib (an EGF inhibitor, R&D Systems, Minneapolis, MN, USA, 200 µM) to the spinal cord and testing continued. In the third group of rats, we applied IL-6 (R&D Systems, Minneapolis, MN, USA, 1 µg/mL) and sIL-6R (R&D Systems, Minneapolis, MN, USA, 1 µg/mL) to the spinal cord for 6 h, then we recorded further responses to stimulation (in the presence of IL-6/sIL-6R), and then we added gefitinib (200 nM) to the trough. The number of action potentials during each stimulus application (15 s) was counted. Mean neuronal responses to a stimulus of successive 30 min time intervals were compared to a 30 min baseline before substance application.

### 4.3. Culture of BV2 Cells and DRG Neurons

We used the well-established murine BV2 microglial cell line (#ATL03001, ICLC, San Martino, Italy, RRID: CVCL_0182 [63,64]). BV2 cells were cultured in DMEM containing 10% heat-inactivated fetal bovine serum (BioWest, Nuaillé, France) and maintained at 37 °C in a humidified incubator fumigated with 5% CO_2_ and air.

DRGs were dissected from mice (C57BL/6J) and collected in Ham’s F12 medium (BioWest, Nuaillé, France). Digestion was followed by collagenase type II (240 U/mL, Sigma, Taufkirchen, Germany) for 1 h and trypsin (10,000 U/mL, Sigma) for 10 min at 37 °C. DRG cells were suspended in Ham’s F12 medium, dispersed by mechanical trituration with a fire-polished Pasteur pipette, and collected by centrifugation (500× *g* for 8 min). Cell pellets were suspended in Ham’s F12 medium and 10% heat-inactivated horse serum (Sigma) supplemented with 1 mM glutamine (Sigma), 1% PenStrep (Thermo Fisher Scientific, Waltham, MA, USA), and 50 ng/mL nerve growth factor (Enzo, Lörrach, Germany). Cells were seeded on Nunclon-12-well-plates (Thermo Fisher) coated with poly-L-Lysine (PLL, 50 µg/mL, Sigma) and placed at 37 °C in a humidified incubator fumigated with 5% CO_2_ and air. Cells were used within 20–30 h of culture.

### 4.4. Stimulation of Cells, SDS-PAGE, and Immunoblotting

BV2 cells and primary DRG neurons from normal mice were starved overnight (BV2 cells) or for 3 h (DRG) in pure DMEM prior to stimulation. For signaling analysis, cells were treated with 25 ng/mL recombinant mouse EGF (ImmunoTools, Friesoythe, Germany) or hyper-IL-6 (25 ng/mL, R&D Systems, Minneapolis, MN, USA), a fusion protein of IL-6 and its soluble IL-6 receptor [36], for different periods of time. In some experiments, stimulation was combined with application of gefitinib (200 nM). Gefitinib inhibits the EGFR family of tyrosine kinases by preventing the binding of ATP to the tyrosine domain of EGFR, thereby blocking EGFR activation [33]. In this case, the inhibitor was preincubated for 30 min and always present during agonist stimulations. The reaction was stopped on ice, the medium was aspirated, and the cells were scraped and harvested with RIPA lysis buffer [20 mM Tris-HCl, 150 mM NaCl, 2.5 mM Na_4_P_2_O_7_, 1 mM EGTA, 1 mM β-glycerophosphate, 1% C_24_H_39_NaO_4_, 1% NP-40, pH 7.4, freshly supplemented with protease inhibitor cocktail tablets (Roche, Mannheim, Germany)]. After a freeze–thaw cycle, cell lysates were centrifuged and Laemmli loading buffer was added to the supernatants. The stimulation experiments were repeated at least three times.

For protein analysis, protein samples were separated on 10% PAGE-gels at 125 V and transferred to a PVDF membrane (Millipore, Billerica, MA, USA). Immunoblotting followed with anti-Phospho-Stat3-Tyr705 (#9145, RRID:AB_2491009), anti-Stat3 (#4904, RRID:AB_331269), anti-Phospho-Erk1/2-Thr202/Tyr204 (#9106, RRID: AB_331768), and anti-Erk1/2 (#9102, RRID: AB_330744). These antibodies were from Cell Signaling Technology, Danvers, MA, USA. Furthermore, we used anti-Gapdh (#G8795, RRID: AB_1078991, Sigma) and anti-vinculin (#V9264, RRID: AB_10603627, Sigma). Signals were visualized with HRP-conjugated secondary antibodies (RRID:AB_2721169 and RRID:AB_2891080, SeraCare, Milford, MA, USA) and an enhanced chemiluminescence reaction (Thermo Fisher Scientific) according to the manufacturer, using a CCD camera system with GeneSnap 7.12 software (RRID: SCR_014249, Synoptics, Cambridge, UK).

### 4.5. Antigen-Induced Arthritis in the Rat (AIA)

For AIA induction, female Lewis rats (aged 6–8 weeks, 170–200 g, Janvier, Le Genest-Saint-Isle, France) were immunized with 500 µg antigen (methylated bovine serum albumin [mBSA]) in saline emulsified with 500 µL complete Freund’s adjuvant (CFA), supplemented with 2 mg/mL Mycobacterium tuberculosis strain H37RA. The antigen was injected twice bilaterally into the back and neck, with a 1-week interval between immunizations. After another 2 weeks, the antigen (sterile mBSA solution, 500 µg in 50 µL) was injected into the cavity of the left knee joint to induce monoarticular AIA. We documented swelling of the knee joint and pain-related behaviors. We scored gait abnormalities: (0) no guarding; (1) transient limping after brief compression of the inflamed knee; (2) persistent, visible, spontaneous limping; (3) no use of the ipsilateral hind limb; and (4) no walking. Mechanical hyperalgesia at the inflamed knee joint (primary mechanical hyperalgesia) and mechanical hyperalgesia at the contralateral knee joint were assessed by applying increasing compression of the knee joint with a digital pressure application measurement (PAM) device until the animals attempted to escape or vocalized. Mechanical hyperalgesia at the plantar aspect of the hind paws (secondary mechanical hyperalgesia) was assessed with a dynamic plantar aesthesiometer. A blunt tip was positioned below the plantar surface, and the force was increased until the paws showed a withdrawal reflex. Secondary thermal hyperalgesia at the plantar aspect of the hind paws was assessed using the Hargreaves Apparatus (Ugo Basile, Gemonio Varese, Italy) [65]. An infrared source was placed under the plantar surface, and latency to heat-evoked paw withdrawal was measured. Asymmetric weight bearing as a functional measure of pain-related guarding of the inflamed hind limb was assessed with an incapacitance tester. The pre-arthritic baseline values before AIA were the values obtained after the second immunization.

### 4.6. Glucose-6-Phosphate-Isomerase (G6PI)-Induced Arthritis of Mice

Female wild-type DBA/1JRj mice (9–12 weeks old, either bred by the Animal Facility of the University Hospital Jena or purchased from Janvier Laboratories) were immunized subcutaneously on day 0 with 400 μg of recombinant human G6PI emulsified 1:1 (volume/volume) in CFA. In one group of mice, regulatory T (Treg) cells were depleted in vivo 11 and 8 days before immunization, with 400 μg of anti-CD25 antibody from hybridoma cell culture. We regularly assessed the cumulative clinical score, taking into account the swelling and erythema of the metatarsal/metacarpal regions and the carpo-metacarpal/tarsometatarsal joints, as well as the number of swollen toes of each paw. The clinical score was graded as 0 = normal; 1 = mild redness and swelling; 2 = moderate swelling; and 3 = severe swelling with edema. In order to quantify bone destruction, high-resolution micro-computed tomography (micro-CT) imaging was performed on formalin-fixed hind paws using a TomoScope Synergy Twin imaging system (TomoScope Development, Erlangen, Germany). Local mechanical and thermal (heat) hyperalgesia (withdrawal threshold) at the hind paws was assessed using a dynamic plantar aesthesiometer for mechanical hyperalgesia and the Hargreaves plantar test for thermal hyperalgesia. For each test, thresholds were averaged from up to 3 consecutive stimuli, and 2 tests prior to immunization defined the baseline. Hind limb gait abnormalities (guarding score) were scored as in rats (see above).

### 4.7. Labeling of EGFR and pEGFR in Rat DRGs and EFGR in BV2 Cells

Rats from the AIA study were deeply anesthetized, transcardially perfused with 4% ice-cold phosphate-buffered paraformaldehyde (PFA), and DRGs from segments L1–L5 of the inflamed sides were excised, embedded in paraffin, cut into 5 µm sections and mounted on slides (for details, see Leuchtweis et al. [31]). Sections from normal control rats and rats at different stages of AIA (immunization control (IC), 3d, 42d, 84d) were deparaffinized in xylol, hydrated, and autoclaved for 15 min (120 °C, 1 bar) in 0.1 M citrate buffer (pH 6.0). After cooling, the sections were washed in PBS and incubated for 30 min in PBS plus 1% Triton-X100 plus 2% goat serum (Rockland, Gilbertsville, PA, USA). The sections were then incubated overnight at 4 °C in a humid chamber with the primary antibody diluted 1:100 in PBS plus 1% Triton X-100 containing 1% cold water fish skin gelatin. Two different anti-EGFR antibodies were used: 1. an antibody against total EGFR (anti-EGFR, #ab52894, RRID: AB_869579, Abcam, Cambridge, UK, knockout validated), and 2. an antibody that detects EGFR only when phosphorylated at tyrosine 1068 (pEGFR, #2234, RRID: AB_331701, Cell Signaling Technology). After overnight incubation, the sections were washed with PBS. Alexa-488 goat anti-rabbit antibody (#A11008, RRID: AB_143165, Thermo Fisher Scientific) was used a as secondary antibody for both antibodies. This antibody was diluted 1:100 in the same buffer as the primary antibodies and administered to the sections for 4 h at 20 °C. After three washes, the sections were embedded in Aqua-Poly/Mount (Polysciences, Warrington, PA, USA). Antibody control experiments were carried out without the primary antibodies.

For analysis, the proportion of neuronal profiles with antibody labeling was determined in every other section using a light microscope (Axioplan 2, Zeiss, Jena, Germany) coupled to an image analysis system (illumination time 100 ms, Axiovision, Zeiss). The mean fluorescence value and radius were determined for each neuron. All neurons were considered positive if they showed a fluorescence value above that of neurons from control incubations, not treated with the primary antibody. The proportions of positive neurons for each antibody from different rats and time points of the AIA were averaged and expressed as mean ± SEM.

BV2 cells were seeded and grown on PLL-coated coverslips, and fixed with 4% PFA for 30 min. The procedure for labeling EGFR in BV2 cells was identical to that for labeling EGFR in DRG neurons in sections.

### 4.8. Labeling of EGFR and pEGFR (Tyr1068) in the Spinal Cord of Mice

Mice from the G6PI-induced arthritis study were sacrificed by cervical dislocation under deep isoflurane (5.0%) anesthesia, and the lumbar spinal cords were excised, embedded in paraffin, cut into 7 µm sections, and mounted on slides (for details see [32]). Sections from mice 6 or 42 days after G6PI-induced arthritis with or without Treg cell depletion and normal control mice were used for this study. Spinal cord sections were handled as described for DRGs except that PBS 1% Triton X-100 and 2% normal goat serum (AURION, Wageningen, The Netherlands), or normal donkey serum (Dianova, Hamburg, Germany) were used for antibody dilution. The following primary antibodies were used: anti-EGFR antibody against total EGFR (#ab52894, rabbit, monoclonal, 1:50, Abcam, RRID: AB_869579), anti-phospho-EGFR-(Y1068) (#ab40815, rabbit, monoclonal, 1:50, Abcam, RRID: AB_732110), anti-Neuron-Specific Enolase (NSE) (#804901, mouse, monoclonal, 1:50, Biolegend, San Diego, CA, USA, RRID: AB_2564673), anti-Iba1 (#GTX89792, goat, polyclonal, 1:50, Genetex, Irvine, CA, USA, RRID: AB_10724185), anti-GFAP (#3670, mouse, monoclonal, 1:500, Cell Signaling, RRID: AB_561049). Secondary antibodies were anti-rabbit Alexa fluor 488 (#A11008, goat, polyclonal, 1:100, Invitrogen, Waltham, MA, USA, RRID: AB_143165), anti-mouse Alexa fluor 568 (#A11011, goat, polyclonal, 1:100, Invitrogen, RRID: AB_2534072), and anti-goat Alexa fluor 488 (#A11055, donkey, polyclonal, Invitrogen, RRID: AB_2534102). The slides were then washed three times in PBS for 10 min. Hoechst 34580 dye (Thermo Fisher) was used at 1:1000 dilution in PBS and incubated for 5 min for nuclear staining. After washing, the slices were embedded in Aqua Poly/Mount (Polysciences).

For analysis, images of spinal cord slices were obtained using confocal laser scanning microscopy (LSM, TCS SP5, Leica, Wetzlar, Germany). Dyes were excited at wave lengths of 405 nm, 488 nm, and 561 nm. Fluorescence signals were detected using a 63 × 1.40 oil objective. For quantification of EGFR expression, LSM stacks were acquired every 1 µm of each sample. Maximum intensity projections were performed and analyzed using ImageJ software (version: 1.54b, NIH, Bethesda, MD, USA). Neurons were selected by double labeling. First, NSE antibody was used to select only the neurons in the spinal cord slices. Then fluorescence signals of pEGFR were measured in these neurons. For 10 randomly selected neurons per section, area, integrated density, and the mean gray value were determined. Corrected total cell fluorescence (CTCF) was measured using the formula [CTCF = integrated density − (area of selected cell × mean fluorescence of background readings)] as described by McCloy et al. [66]. The average CTCF of 10 cells represents one animal.

### 4.9. Statistics

Data in line and bar graphs are presented as mean ± SEM or percentages. Scatter plots show the distribution of individual data points. The statistics were calculated using SPSS Statistics 24 (RRID: SCR_019096, IBM, Armonk, NY, USA) and GraphPad Prism (RRID: SCR_002798, San Diego, CA, USA) software. Normality of the data was tested with the Kolmogorov–Smirnov test, and the data were further analyzed with parametric and non-parametric tests, respectively, including the Bonferroni correction. Applied statistical calculations are indicated in each figure legend. Significance was accepted at *p* ≤ 0.05.

## Data Availability

Data will be made available on request.

## Supplementary Materials

The following supporting information can be downloaded at: https://www.mdpi.com/article/10.3390/ijms25137168/s1.

## References

[1] Dayer J.M., Choy E. (2010). Therapeutic targets in rheumatoid arthritis: The interleukin-6 receptor. Rheumatology.

[2] Fonseca J.E., Santos M.J., Canhao H., Choy E. (2009). Interleukin-6 as a key player in systemic inflammation and joint destruction. Autoimmun. Rev..

[3] Latourte A., Cherifi C., Maillet J., Ea H.K., Bouaziz W., Funck-Brentano T., Cohen-Solal M., Hay E., Richette P. (2017). Systemic inhibition of IL-6/Stat3 signalling protects against experimental osteoarthritis. Ann. Rheum. Dis..

[4] Park J.Y., Pillinger M.H. (2007). Interleukin-6 in the pathogenesis of rheumatoid arthritis. Bull. NYU Hosp. Jt. Dis..

[5] Smolen J.S., Aletaha D. (2011). Interleukin-6 receptor inhibition with tocilizumab and attainment of disease remission in rheumatoid arthritis: The role of acute-phase reactants. Arthritis Rheum.

[6] Wiegertjes R., van de Loo F.A.J., Blaney Davidson E.N. (2020). A roadmap to target interleukin-6 in osteoarthritis. Rheumatology.

[7] Eitner A., Hofmann G.O., Schaible H.-G. (2017). Mechanisms of Osteoarthritic Pain. Studies in Humans and Experimental Models. Front. Mol. Neurosci..

[8] Eitner A., Pester J., Vogel F., Marintschev I., Lehmann T., Hofmann G.O., Schaible H.-G. (2017). Pain sensation in human osteoarthritic knee joints is strongly enhanced by diabetes mellitus. Pain.

[9] Radojcic M.R., Thudium C.S., Henriksen K., Tan K., Karlsten R., Dudley A., Chessell I., Karsdal M.A., Bay-Jensen A.C., Crema M.D. (2017). Biomarker of extracellular matrix remodelling C1M and proinflammatory cytokine interleukin 6 are related to synovitis and pain in end-stage knee osteoarthritis patients. Pain.

[10] Andratsch M., Mair N., Constantin C.E., Scherbakov N., Benetti C., Quarta S., Vogl C., Sailer C.A., Üceyler N., Brockhaus J. (2009). A key role for gp130 expressed on peripheral sensory nerves in pathological pain. J. Neurosci..

[11] Brenn D., Richter F., Schaible H.-G. (2007). Sensitization of unmyelinated sensory fibers of the joint nerve to mechanical stimuli by interleukin-6 in the rat: An inflammatory mechanism of joint pain. Arthritis Rheum..

[12] Dina O.A., Green P.G., Levine J.D. (2008). Role of interleukin-6 in chronic muscle hyperalgesic priming. Neuroscience.

[13] Kawasaki Y., Zhang L., Cheng J.K., Ji R.R. (2008). Cytokine mechanisms of central sensitization: Distinct and overlapping role of interleukin-1beta, interleukin-6, and tumor necrosis factor-alpha in regulating synaptic and neuronal activity in the superficial spinal cord. J. Neurosci..

[14] König C., Morch E., Eitner A., Möller C., Turnquist B., Schaible H.-G., Ebersberger A. (2016). Involvement of Spinal IL-6 Trans-Signaling in the Induction of Hyperexcitability of Deep Dorsal Horn Neurons by Spinal Tumor Necrosis Factor-Alpha. J. Neurosci..

[15] Vazquez E., Kahlenbach J., Segond von Banchet G., König C., Schaible H.-G., Ebersberger A. (2012). Spinal interleukin-6 is an amplifier of arthritic pain in the rat. Arthritis Rheum..

[16] Arruda J.L., Colburn R.W., Rickman A.J., Rutkowski M.D., DeLeo J.A. (1998). Increase of interleukin-6 mRNA in the spinal cord following peripheral nerve injury in the rat: Potential role of IL-6 in neuropathic pain. Brain Mol. Brain Res..

[17] Bao L., Zhu Y., Elhassan A.M., Wu Q., Xiao B., Zhu J., Lindgren J.U. (2001). Adjuvant-induced arthritis: IL-1 beta, IL-6 and TNF-alpha are up-regulated in the spinal cord. Neuroreport.

[18] Boettger M.K., Leuchtweis J., Kümmel D., Gajda M., Bräuer R., Schaible H.-G. (2010). Differential effects of locally and systemically administered soluble glycoprotein 130 on pain and inflammation in experimental arthritis. Arthritis Res. Ther..

[19] DeLeo J.A., Colburn R.W., Nichols M., Malhotra A. (1996). Interleukin-6-mediated hyperalgesia/allodynia and increased spinal IL-6 expression in a rat mononeuropathy model. J. Interf. Cytokine Res..

[20] Nowell M.A., Richards P.J., Horiuchi S., Yamamoto N., Rose-John S., Topley N., Williams A.S., Jones S.A. (2003). Soluble IL-6 receptor governs IL-6 activity in experimental arthritis: Blockade of arthritis severity by soluble glycoprotein 130. J. Immunol..

[21] Rose-John S. (2012). IL-6 trans-signaling via the soluble IL-6 receptor: Importance for the pro-inflammatory activities of IL-6. Int. J. Biol. Sci..

[22] Ray K., Ujvari B., Ramana V., Donald J. (2018). Cross-talk between EGFR and IL-6 drives oncogenic signaling and offers therapeutic opportunities in cancer. Cytokine Growth Factor Rev..

[23] Wang Y., van Boxel-Dezaire A.H., Cheon H., Yang J., Stark G.R. (2013). STAT3 activation in response to IL-6 is prolonged by the binding of IL-6 receptor to EGF receptor. Proc. Natl. Acad. Sci. USA.

[24] Borges J.P., Mekhail K., Fairn G.D., Antonescu C.N., Steinberg B.E. (2021). Modulation of Pathological Pain by Epidermal Growth Factor Receptor. Front. Pharmacol..

[25] Tavassoly O., Sato T., Tavassoly I. (2020). Inhibition of Brain Epidermal Growth Factor Receptor Activation: A Novel Target in Neurodegenerative Diseases and Brain Injuries. Mol. Pharmacol..

[26] Andres C., Meyer S., Dina O.A., Levine J.D., Hucho T. (2010). Quantitative automated microscopy (QuAM) elucidates growth factor specific signalling in pain sensitization. Mol. Pain.

[27] Martin L.J., Smith S.B., Khoutorsky A., Magnussen C.A., Samoshkin A., Sorge R.E., Cho C., Yosefpour N., Sivaselvachandran S., Tohyama S. (2017). Epiregulin and EGFR interactions are involved in pain processing. J. Clin. Investig..

[28] Romano R., Bucci C. (2020). Role of EGFR in the Nervous System. Cells.

[29] Li Z.W., Tang R.H., Zhang J.P., Tang Z.P., Qu W.S., Zhu W.H., Li J.J., Xie M.J., Tian D.S., Wang W. (2011). Inhibiting epidermal growth factor receptor attenuates reactive astrogliosis and improves functional outcome after spinal cord injury in rats. Neurochem. Int..

[30] Erschbamer M., Pernold K., Olson L. (2007). Inhibiting epidermal growth factor receptor improves structural, locomotor, sensory, and bladder recovery from experimental spinal cord injury. J. Neurosci..

[31] Leuchtweis J., Segond von Banchet G., Eitner A., Ebbinghaus M., Schaible H.-G. (2020). Pain-related behaviors associated with persistence of mechanical hyperalgesia after antigen-induced arthritis in rats. Pain.

[32] Ebbinghaus M., Müller S., Segond von Banchet G., Eitner A., Wank I., Hess A., Hilger I., Kamradt T., Schaible H.-G. (2019). Contribution of Inflammation and Bone Destruction to Pain in Arthritis: A Study in Murine Glucose-6-Phosphate Isomerase-Induced Arthritis. Arthritis Rheumatol..

[33] Roskoski R. (2019). Small molecule inhibitors targeting the EGFR/ErbB family of protein-tyrosine kinases in human cancers. Pharmacol. Res..

[34] Hsu M.P., Frausto R., Rose-John S., Campbell I.L. (2015). Analysis of IL-6/gp130 family receptor expression reveals that in contrast to astroglia, microglia lack the oncostatin M receptor and functional responses to oncostatin M. Glia.

[35] Ferrer I., Alcantara S., Ballabriga J., Olive M., Blanco R., Rivera R., Carmona M., Berruezo M., Pitarch S., Planas A.M. (1996). Transforming growth factor-alpha (TGF-alpha) and epidermal growth factor-receptor (EGF-R) immunoreactivity in normal and pathologic brain. Prog. Neurobiol..

[36] Fischer M., Goldschmitt J., Peschel C., Brakenhoff J.P., Kallen K.J., Wollmer A., Grötzinger J., Rose-John S. (1997). A bioactive designer cytokine for human hematopoietic progenitor cell expansion. Nat. Biotechnol..

[37] Frey O., Reichel A., Bonhagen K., Morawietz L., Rauchhaus U., Kamradt T. (2010). Regulatory T cells control the transition from acute into chronic inflammation in glucose-6-phosphate isomerase-induced arthritis. Ann. Rheum. Dis..

[38] Schubert D., Maier B., Morawietz L., Krenn V., Kamradt T. (2004). Immunization with glucose-6-phosphate isomerase induces T cell-dependent peripheral polyarthritis in genetically unaltered mice. J. Immunol..

[39] Zhang Z., Ma Z., Zou W., Guo H., Liu M., Ma Y., Zhang L. (2019). The Appropriate Marker for Astrocytes: Comparing the Distribution and Expression of Three Astrocytic Markers in Different Mouse Cerebral Regions. BioMed Res. Int..

[40] König C., Vazquez E., Eß S., Ebbinghaus M., Vorpahl B., Ebersberger A., Schaible H.-G. (2021). Spinal interleukin-1beta induces mechanical spinal hyperexcitability in rats: Interactions and redundancies with TNF and IL-6. J. Neurochem..

[41] Huerta J.J., Diaz-Trelles R., Naves F.J., Llamosas M.M., Del Valle M.E., Vega J.A. (1996). Epidermal growth factor receptor in adult human dorsal root ganglia. Anat. Embryol..

[42] Wang S., Liu S., Xu L., Zhu X., Liu W., Tian L., Chen Y., Wang Y., Nagendra B.V.P., Jia S. (2019). The upregulation of EGFR in the dorsal root ganglion contributes to chronic compression of dorsal root ganglions-induced neuropathic pain in rats. Mol. Pain.

[43] Bakker J., Spits M., Neefjes J., Berlin I. (2017). The EGFR odyssey—From activation to destruction in space and time. J. Cell Sci..

[44] Verma V., Khoury S., Parisien M., Cho C., Maixner W., Martin L.J., Diatchenko L. (2020). The dichotomous role of epiregulin in pain. Pain.

[45] Wetzker R., Böhmer F.D. (2003). Transactivation joins multiple tracks to the ERK/MAPK cascade. Nat. Rev. Mol. Cell Biol..

[46] Ebbinghaus M., Segond von Banchet G., Massier J., Gajda M., Bräuer R., Kress M., Schaible H.-G. (2015). Interleukin-6-dependent influence of nociceptive sensory neurons on antigen-induced arthritis. Arthritis Res. Ther..

[47] Chen J., Zeng F., Forrester S.J., Eguchi S., Zhang M.Z., Harris R.C. (2016). Expression and Function of the Epidermal Growth Factor Receptor in Physiology and Disease. Physiol. Rev..

[48] Wong R.W., Guillaud L. (2004). The role of epidermal growth factor and its receptors in mammalian CNS. Cytokine Growth Factor Rev..

[49] Li Z.W., Zhao J.J., Li S.Y., Cao T.T., Wang Y., Guo Y., Xi G.J. (2021). Blocking the EGFR/p38/NF-kappaB signaling pathway alleviates disruption of BSCB and subsequent inflammation after spinal cord injury. Neurochem. Int..

[50] Yamada M., Ikeuchi T., Hatanaka H. (1997). The neurotrophic action and signalling of epidermal growth factor. Prog. Neurobiol..

[51] Ozturk A.M., Sozbilen M.C., Sevgili E., Dagci T., Özyalcin H., Armagan G. (2018). Epidermal growth factor regulates apoptosis and oxidative stress in a rat model of spinal cord injury. Injury.

[52] Zhang S., Ju P., Tjandra E., Yeap Y., Owlanj H., Feng Z. (2016). Inhibition of Epidermal Growth Factor Receptor Improves Myelination and Attenuates Tissue Damage of Spinal Cord Injury. Cell. Mol. Neurobiol..

[53] Abe K., Xie F.J., Saito H. (1991). Epidermal growth factor enhances short-term potentiation and facilitates induction of long-term potentiation in rat hippocampal slices. Brain Res..

[54] Abe K., Saito H. (1992). Epidermal growth factor selectively enhances NMDA receptor-mediated increase of intracellular Ca^2+^ concentration in rat hippocampal neurons. Brain Res..

[55] Terlau H., Seifert W. (1989). Influence of epidermal growth factor on long-term potentiation in the hippocampal slice. Brain Res..

[56] Schaible H.-G., König C., Ebersberger A. Spinal pain processing in arthritis: Neuron and glia (inter)actions. J. Neurochem..

[57] Lin S.Y., Makino K., Xia W., Matin A., Wen Y., Kwong K.Y., Bourguignon L., Hung M.C. (2001). Nuclear localization of EGF receptor and its potential new role as a transcription factor. Nat. Cell Biol..

[58] Lo H.W., Ali-Seyed M., Wu Y., Bartholomeusz G., Hsu S.C., Hung M.C. (2006). Nuclear-cytoplasmic transport of EGFR involves receptor endocytosis, importin beta1 and CRM1. J. Cell. Biochem..

[59] Lo H.W., Hsu S.C., Ali-Seyed M., Gunduz M., Xia W., Wei Y., Bartholomeusz G., Shih J.Y., Hung M.C. (2005). Nuclear interaction of EGFR and STAT3 in the activation of the iNOS/NO pathway. Cancer Cell.

[60] Wang S.C., Lien H.C., Xia W., Chen I.F., Lo H.W., Wang Z., Ali-Seyed M., Lee D.F., Bartholomeusz G., Ou-Yang F. (2004). Binding at and transactivation of the COX-2 promoter by nuclear tyrosine kinase receptor ErbB-2. Cancer Cell.

[61] Turnquist B., Leverentz M., Swanson E. (2004). Neural spike classification using parallel selection of all algorithm parameters. J. Neurosci. Methods.

[62] Forster C., Handwerker H.O. (1990). Automatic classification and analysis of microneurographic spike data using a PC/AT. J. Neurosci. Methods.

[63] Blasi E., Barluzzi R., Bocchini V., Mazzolla R., Bistoni F. (1990). Immortalization of murine microglial cells by a v-raf/v-myc carrying retrovirus. J. Neuroimmunol..

[64] Henn A., Lund S., Hedtjarn M., Schrattenholz A., Porzgen P., Leist M. (2009). The suitability of BV2 cells as alternative model system for primary microglia cultures or for animal experiments examining brain inflammation. ALTEX.

[65] Hargreaves K., Dubner R., Brown F., Flores C., Joris J. (1988). A new and sensitive method for measuring thermal nociception in cutaneous hyperalgesia. Pain.

[66] McCloy R.A., Rogers S., Caldon C.E., Lorca T., Castro A., Burgess A. (2014). Partial inhibition of Cdk1 in G 2 phase overrides the SAC and decouples mitotic events. Cell Cycle.

