# Supplementary material for: Impact of Interleukin-6 Activation and Arthritis on Epidermal Growth Factor Receptor (EGFR) Activation in Sensory Neurons and the Spinal Cord
Source: Int J Mol Sci. 2024 Jun 28;25(13):7168. doi: 10.3390/ijms25137168 (PMC11241234; doi:10.3390/ijms25137168)
Supplement: Supplementary file 1 [file ijms-25-07168-s001.zip › ijms-3075910-supplementary.pdf]

Supplementary Figure S1

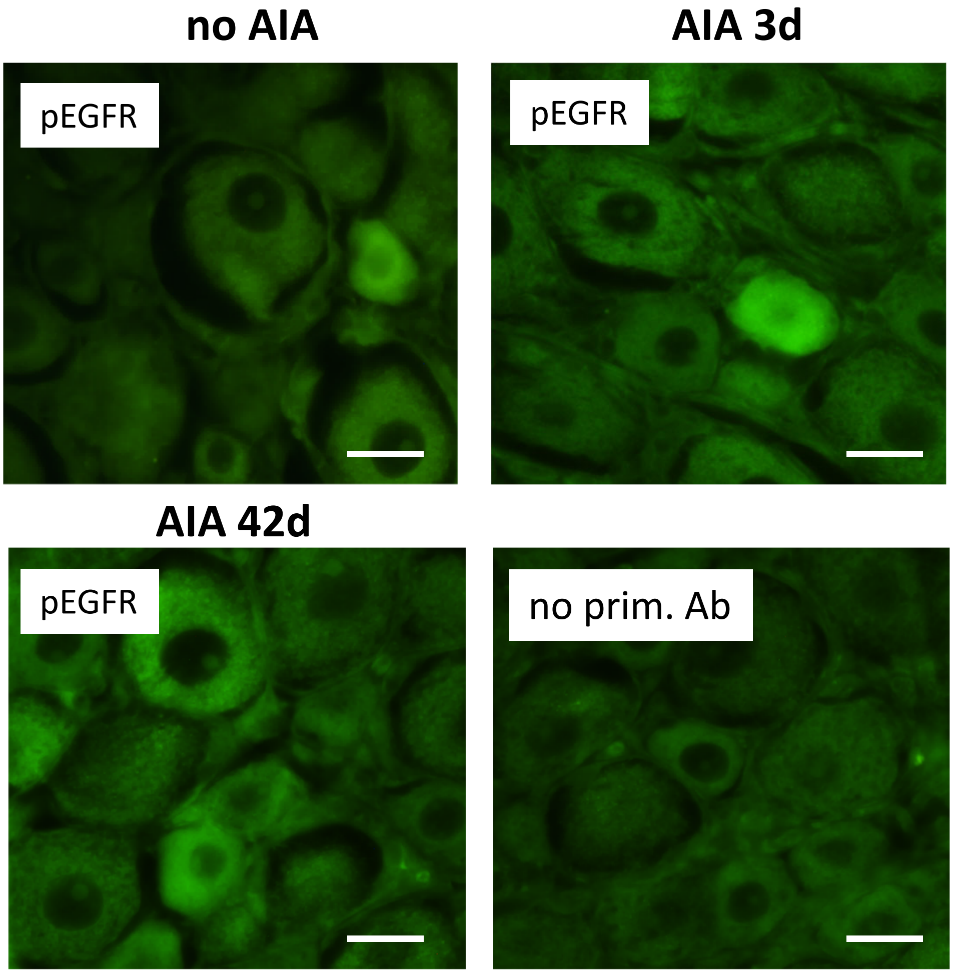

Immunostaining of pEGFR in DRG neurons of normal rats and of rats with AIA. Representative specimens showing pEGFR labeling in DRG sections of heathy rat without AIA, of rat at day 3 of AIA, of rats at day 42 of AIA, and of a control section after omission of the antibody against pEGFR. Scale bar 20 µm.

Supplementary Figure S2

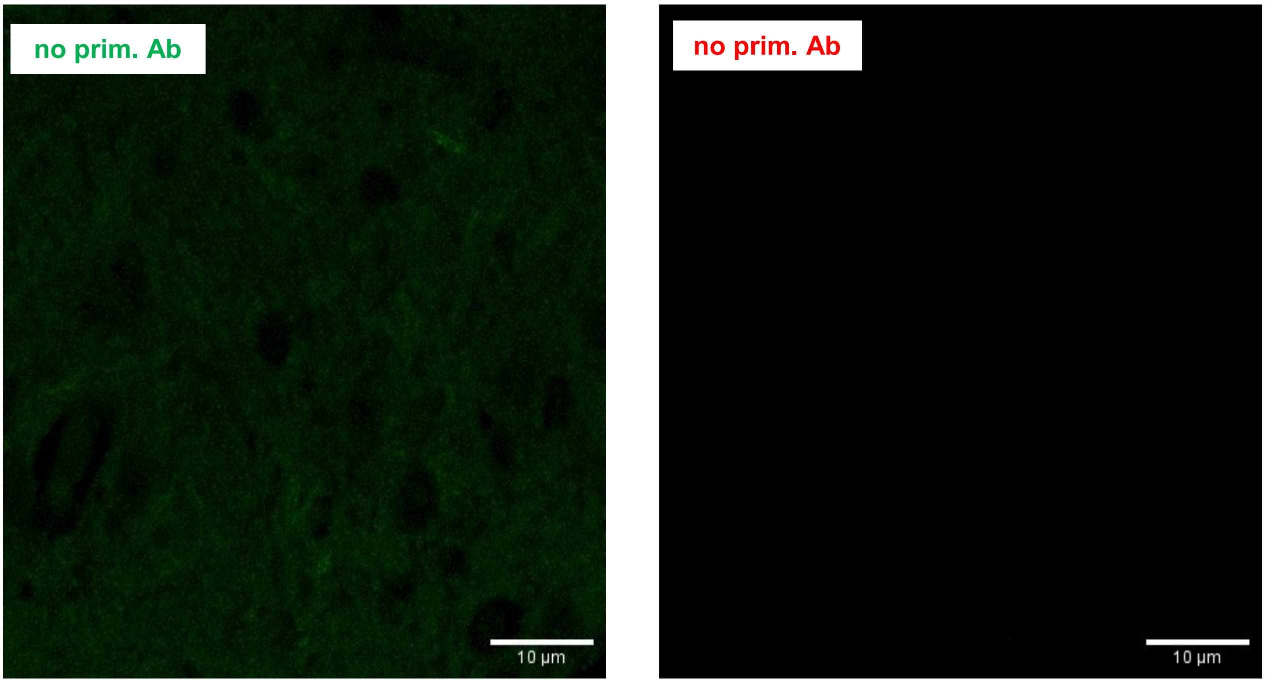

Control immunostainings with omission of primary antibodies of murine spinal cord tissues from G6PI arthritic mice at day 42. Control stainings accompined the immunohistochemistry of pEGFR (green, left) and NSE (red, right).
